# Thermotherapy. An alternative for the treatment of American cutaneous leishmaniasis.

**DOI:** 10.1186/1745-6215-13-58

**Published:** 2012-05-17

**Authors:** Liliana López, Martha Robayo, Margarita Vargas, Iván D Vélez

**Affiliations:** 1 Program for the Study and Control of Tropical Disease, University of Antioquia, Carrera 53 #61-30, Medellín, Colombia; 2 Dirección de Sanidad, DISAN, Colombia Army, Bogotá, Colombia

**Keywords:** Thermotherapy, American cutaneous leishmaniasis, meglumine antimoniate, treatment

## Abstract

**Background:**

Pentavalent antimonials (Sb^5^) and miltefosine are the first-line drugs for treating cutaneous leishmaniasis in Colombia; however, toxicity and treatment duration negatively impact compliance and cost, justifying an active search for better therapeutic options. We compared the efficacy and safety of thermotherapy and meglumine antimoniate for the treatment of cutaneous leishmaniasis in Colombia.

**Method:**

An open randomized Phase III clinical trial was performed in five military health centres. located in northwestern, central and southern Colombia. Volunteers with parasitological positive diagnosis (Giemsa-stained smears) of cutaneous leishmaniasis were included. A single thermotherapy session involving the application of 50°C at the center and active edge of each lesion. Meglumine antimoniate was administered intramuscularly at a dose of 20 mg Sb^5^/kg weight/day for 20 days.

**Results:**

Both groups were comparable. The efficacy of thermotherapy was 64% (86/134 patients) by protocol and 58% (86/149) by intention-to-treat. For the meglumine antimoniate group, efficacy by protocol was 85% (103/121 patients) and 72% (103/143) by intention-to-treat, The efficacy between the treatments was statistically significant (p 0.01 and <0.001) for analysis by intention to treat and by protocol, respectively. There was no difference between the therapeutic response with either treatment regardless of the *Leishmania* species responsible for infection. The side effects of meglumine antimoniate included myalgia, arthralgia, headache and fever. Regarding thermotherapy, the only side effect was pain at the lesion area four days after the initiation of treatment.

**Conclusion:**

Although the efficacy rate of meglumine antimoniate was greater than that of thermotherapy for the treatment of cutaneous leishmaniasis, the side effects were also greater. Those factors, added to the increased costs, the treatment adherence problems and the progressive lack of therapeutic response, make us consider thermotherapy as a first line treatment for cutaneous leishmaniasis. Registered ClinicalTrials.gov NCT00471705

## Background

Leishmaniasis is a group of diseases caused by parasites from the *Leishmania* genus. It is transmitted by female insects from the *Lutzomyia* genus in America and Phlebotomus in the Old World, affecting humans and domestic and wild mammals [1,2]. It is endemic in more than 98 countries. There are 2 million new cases per year, of which 1.5 million present in the cutaneous form [3].

In Colombia, there has been a reappearance of leishmaniasis. During the years of 2005–2008, 61,120 new cases were diagnosed. Among these cases, 34,262 (56.1%) occurred in National Army soldiers Statistical data (Dirección de Sanidad del Ejército (DISAN)).

Since the 1940s, the pentavalent antimonials (Sb^5^) (meglumine antimoniate and sodium stibogluconate) have been considered first line medications for the treatment of leishmaniasis. In Colombia, health authorities recommend a 20 mg Sb^5^/kg/day dose for 20 days to treat cutaneous leishmaniasis and 28 days of therapy to treat mucosal leishmaniasis (ML) and visceral leishmaniasis (VL) [4].

However, the pentavalent antimonials are expensive (approximately $200 per patient), cardiotoxic (P wave prolongation, inverted T waves, ST elevation and QT prolongation), hepatotoxic (liver enzymes elevation), nephrotoxic (BUN and creatinine elevation) and can also cause pancreatitis, leukopenia, thrombocytopenia, arthralgia and myalgia.

These complications, in combination with the parenteral route and the duration of treatment, cause adherence problems. Antimonials are contraindicated during pregnancy and lactation, in very young children, in patients with hypersensitivity to the drug and in patients suffering from chronic conditions. Some reports have shown a decrease in the sensitivity of Leishmania parasites to antimonials [3-13]. In the Old World leishmaniasis caused by *L. (L) tropica,* the parasite resistance is the main problem for the use of antimony [14,15]. Therefore, all of the factors mentioned above reinforce the need for alternative therapies.

Several oral medications have been tested for the treatment of CL, such as dapsone [16], ketoconazol [17,18], mefloquine [19], allopurinol [20], miltefosine [21-25] and others [4], none of which are completely effective. Comparisons of the efficacy of different drugs are complicated by the different protocols used in each study. According to a recent meta-analysis of clinical trials for CL, this lack of standardization of methodologies makes it impossible to obtain conclusive information [4,26,27].

Some studies have shown that it is possible to induce healing of lesions by treatment with local heat [28-30]. Ethnomedical studies have shown that, in rural communities of South America and Africa, the empiric application of caustic materials (powder, hot brown sugar, silver nitrate, oil, battery) or the cauterization of the lesions with hot metal objects (spoons, knifes) is very common [31-33]. Currently, we employ the local application of an instrument, maintained at 50°C, called Thermomed®. This equipment was used in this study [34].

In addition to the benefits of local treatment, the cost of thermotherapy is significantly lower than the pentavalent antimony. The antimony cost is approximately USD 37.2, which must be added on to the patients disability, medical test before, during and after treatment, and in cases where it is needed, the electrocardiogram. According to calculations by dermatologist of the study, the total cost of local heat therapy, does not exceed USD 20 [3].

The objective of this study was to evaluate the efficacy and safety of Thermotherspy compared with Meglumine antimoniate for the treatment of Cutaneous Leishmnaniosis in Colombia.

## Materials and methods

### Study design

We conducted an open randomized, phase III clinical trial to compare the differences in efficacy and safety between thermotherapy and meglumine antimoniate.

### Population and site of the study

The study was conducted between June 2006 and April 2008. The subjects were adult males enlisted in the Colombian Army. The study took place in five military health clinics in the northeast, south and central regions of Colombia.

### Inclusion Criteria

Patients included in this study met the following criteria: a) positive parasitologic diagnosis of leishmaniasis, b) no previous treatment for this parasitic infection, c) laboratory exams including renal, hepatic and hematologic testing and d) voluntary agreement to participate in the study.

### Exclusion criteria

Patients with the following characteristics were excluded: a) chronic concomitant diseases, b) lesions compromising the mucosa, c) presence of 10 or more cutaneous lesions with a negative Montenegro test or d) cutaneous lesions located less than 2 cm from the nasal or oral mucosa, eyes or near the anal or urogenital orifices.

### Interventions

Thermotherapy (Thermomed®, Thermosurgery Inc. Phoenix-USA). Following the aseptic preparation of the lesions and local anesthesia with 2% xylocaine, we applied a single session of thermotherapy to the center, active borders and peripheral area of the lesions. Each thermal application was at 50°C and lasted for 30 seconds; the number of applications depended on the size of the lesion. After the thermotherapy session and over the next 10 days, an antibiotic ointment (fusidic acid) was applied over the lesions, which were then covered with sterile gauze to prevent secondary infections. Meglumine antimoniate (Glucantime®, Aventis, Paris, France) was administered intramuscularly under medical supervision at a dose of 20 mg Sb^5^/kg/day for 20 days. All patients of both groups remained within the military unit for the duration of the treatment.

### Data collection and samples

After providing writing informed consent, all patients were given a medical chart with pertinent demographic information, lesion data and a review of inclusion/exclusion criteria. We subsequently photographed each lesion. Histologic samples were taken from all patients to confirm the diagnosis and to identify the type of Leishmania using PCR-RFLP, according to published guidelines [35].

Clinical samples were taken from all subjects for the parasitological confirmation of leishmaniasis by direct microscopic examination, lesion aspirate samples were taken from each of the patients and were processed as explained in other publications [35,36]. In brief, the aspirates were cultured in NNN culture medium, incubated at 26°C, and from the fourth day on they were observed weekly for one month, in the inverted microscope in search of promastigotes. The media cultures were labeled with the code of each participant and were stored on independent racks to avoid mixing. Each week positive cultures were mass cultured in 50 ml glass bottles with NNN modified medium, one part was frozen in liquid nitrogen and is now stored in the PECET Criobank, and the other was used for species identification; for the DNA extraction, Promastigotes cultures were centrifuged and washed three times for 5 min at 1000 g in PBS pH 7.6. Parasites were then re-suspended in 200 μl of lysis buffer (10 mM Tris–HCl, pH 8,5 mM EDTA, 0.5% SDS, 200 mM NaCl, 100 μg/ml proteinase K) for 90 min at 65°C with moderate agitation. DNA was precipitated by adding two volumes of absolute ethanol to the lysate and the solution was mixed by inversion, and centrifuged for 15 min at 10,000 g. To recover the DNA, the supernatant was discarded and the pellet was dried for 15 min at 65°C and then re-suspended in 200 μl of water or TE. Then for the PCR amplification of the *Cpb gene.* Due to the partial degree of variability of the *Cpb* sequence between the *Viannia* and *Leishmania* subgenus, *Cpb-*specific primers were designed using the primer3plus software (http://www.bioinformatics.nl/cgibin/primer3plus/primer3plus.cg). A region of the Cpb gene in *L. (V) panamensis, L. (V) braziliensis* and *L. (V) guyanensis* were amplified with the primers FwNTerBra (5´-ATGACGGTGCCGAGGGTCCT-3´) and RvCTerBra (5’-CTACTTGAACGTGCAGAT-3’); while primers FwRGPS (5’-ATGGCGACGTCGAGGGCC-3’) and RvRGPS (5’-CAGGTGTTCATGATCGAGCCC-3’) were used for *L. (L) mexicana* and *L. (L) infantum*; To amplify the *Cpb* region of *L. (L) amazonensis* the primers FwRPAMZ (5’-GGATCCATACACGTGGGCACGCCG-3’) and RvRPAMZ (5’-AAGCTTCTACGTGTAGTGACAGGT-3’) were used. These last pair of primers also amplify for *L. (L)Mexicana.* After each PCR round, 5 μl of the product were separated in a 1% agarose gel and stained with 0,5 μg/ml of ethidium bromide. DNA products were visualized using a Chemidoc image analyzer system (Bio-Rad, Hercules, California, USA).

In order to identify the *Leishmania* species by RFLP, the NEBcutter V2.0 programme (New England Biolab, Ipswich, Massachusetts, USA) was used to generate species-specific restriction sites. To differentiate *L. (V) panamensis* from *L. (V) braziliensis,* 10 μl of the amplification product was digested with *Kpn*I (Promega), the mix was incubated for 6 h at 37°C. To differentiate *L. (V) panamensis* from *L. (V) guyanensis,* 10 μl of the amplification product was digested with *Nsi*I (New England Biolab), this mix was incubated for 16 h at 37°C. To differentiate *L. (L) mexicana* from *L. (L) amazonensis,* 10 μl of the amplification product was digested, with *Nar*I (New England Biolab), and the mix was incubated for 6 h at 37°C. To differentiate *L. (L) mexicana* from *L. (L) infantum,* 10 μl of the amplification product was digested with *Kpn*I (Promega). After digestion, products were separated in 1% or 2% agarose gel using the same conditions described above for visualizing the PCR-*Cpb* amplification products[37,38].

#### Assignment to the treatment group

Subjects were assigned randomly to treatment groups using a generated list in blocks of eight (EpiInfo 3.1). Only the clinical coordinator of the study had access to the list and was in charge of allocating treatments.

#### Follow-up and outcomes

Subjects receiving meglumine antimoniate were evaluated at the beginning and end of treatment, at 6 weeks and at 3 and 6 months after completing the treatment. Subjects receiving thermotherapy were evaluated on the same day they started the treatment, at days 10 and 20, at 6 weeks and at 3 and 6 months after the application of heat, although thermotherapy was applied in an only session the followup of the healing process of the lesions was carried out three weeks after application, coinciding with the visit of final treatment of the group that received meglumine antimoniate. All patients underwent renal, hepatic, pancreatic and hematologic blood function tests before, during and after the completion of treatment. The assessment of side effects was done according to the Common Terminology Criteria for Adverse Events v.3 (CTCAE)[39].

The treatment response was evaluated clinically. For each lesion, the following definitions were used:

#### Initial healing

Complete reepithelialization of all ulcers and complete loss of induration up to three months after the end of treatment.

#### Definitive healing

Initial healing criteria without any recurrence six months after the completion of treatment.

#### Failure

Increase in size of a lesion of more than 50% at the end of treatment or an absence of clinical response at 6 weeks (lesion size reduction less than 50% compared to the evaluation at the end of treatment), signs of lesion activity 3 months after the completion of treatment or recurrence or presence of mucosal leishmaniasis at any time 6 months after the end of treatment.

#### Recurrence

Lesion reactivation in the original site after cicatrization.

#### Reinfection

Presence of new lesions at an anatomic site different from the original lesions after the patient was considered healed and after returning to endemic areas.

In all subjects that had a treatment failure, meglumine antimoniate was provided as rescue therapy at a dose of 20 mg Sb^5^/kg/day for 20 days, according to the Colombian Ministry of Health guidelines [40].

#### Sample size calculation

The sample size was calculated assuming a thermotherapy efficacy of 78%, meglumine antimoniate efficacy of 90%, confidence interval (CI) of 95% and power of 80% (non-inferiority trial). To this sample size number, we added 20% to account for possible loss of subjects to follow-up. The total sample size was 144 subjects per group for a total of 288 participants.

#### Statistical analysis

Data entry and data analysis were performed using ACCESS and SPSS v.15, respectively. The baseline characteristics of volunteers were categorized and analyzed by treatment group. The treatment efficacy was calculated by intention-to-treat and by protocol. The relative risk was calculated using 2x2 tables. For hypothesis tests in dichotomic variables, we used the Fisher's Test or *X*^*2*^ test. The student's *t*-test or the “U of Mann–Whitney” test was used to analyze continuous data. Potential confounding factors and interactions were controlled with analysis stratified by parasite species, number, location, type of lesions and geographic area where the infection occurred. Statistical survival analysis was performed using the Kaplan-Meier method and the log rank test to compare the healing time between both groups. A p value of <0.05 was considered as stastically significant and CI of 95% was used for data analysis.

## Results

We included 292 subjects in the study and randomly assigned 149 to receive thermotherapy and 143 to receive meglumine antimoniate. In the thermotherapy group, two subjects did not want to participate (1%), 13 (9%) did not complete the six month follow-up and a total of 134 (90%) completed the study. In the meglumine antimoniate group, 18 (13%) did not complete the six month follow-up, 2 (1%) withdrew from the Army before completing the study, 2 (1%) died in combat and 121 (85%) completed the study, as shown in Figure 1.

### Recurrences

Recurrence was reported in 6 (4.1%) and 4 (3%) patients for the thermotherapy and meglumine antimoniate groups, respectively. All of these patients received meglumine antimoniate as a rescue treatment, and only one patient, who was assigned to the meglumine antimoniate group, required a third treatment with amphotericine B. All recurrences occurred within 3 months of treatment completion.

### Baseline analysis

As shown in Table 1, both groups had similar demographic, clinical and parasitologic characteristics.

### Therapeutic response

#### Initial healing

Three months after the end of treatment, 64% (95% IC 56–72) of the thermotherapy group and 78% (95% IC 71–85) of the meglumine antimoniate group were healed.

#### Definitive healing

A total of 7 patients (4.8%) from the thermotherapy group and 7 (5%) from the meglumine antimoniate group did not complete follow-up with the 3- and 6-month evaluations. By protocol, 64% (95% IC 55–73) of patients in the thermotherapy group and 85% (95% IC 64–80) of patients in the meglumine antimoniate group showed definitive healing. By intention-to-treat, the definitive healing rate in the thermotherapy group was 58.5% (95% IC 49–66) and 72% (95% IC 78–92) in the meglumine antimoniate group, as shown on Table 2. The efficacy analysis between both treatments showed a statistically significant difference, for the analysis by protocol the meglumine antimoniate is 0.33 times more effective than thermotherapy (RR 1.33 (IC 95% 1.15 – 1.54) p <0.001), when analysis was carried out by intention to treat, the superiority of the meglumine antimoniate maintained, being 0.25 more effective than thermotherapy (RR 1.25 (IC 95% 1.05 – 1.48) p <0.001).

### Group analysis

We identified Leishmania species as agents of infection in 167 patients. In the group treated with meglumine antimoniate, 32 patients (38%) had lesions caused by *L. (V) panamensis* and 52 (62%) by *L. (V) brazililensis.* In the thermotherapy group, 24 patients (29%) had lesions caused by *L. (V) panamensis* and 59 (71%) by *L. (V) brazililensis.*

The healing response in the meglumine antimoniate group, among patients with *L. (V) panamensis* and *L. (V) braziliensis,* was of 72% and 65%, respectively. In the thermotherapy group, the healing response was 58% for *L. (V) panamensis* and 53% for *L. (V) braziliensis.* There was no association found between the treatment (meglumine antimoniate, p = 0.5, and thermotherapy, p = 0.6) and the *Leishmania* species identified.

There was also no association between treatment and other variables such as number, location and lesion type or with the geographical area of Colombia where the infection occurred (Table 3).

### Safety

Table 4 shows the systemic and local side effects found in this study. Frequency and severity of side effects were greater in the meglumine antimoniate group. These side effects occurred during and after treatment. These side effects included fever, myalgia, arthralgia and headache. In the group treated with thermotherapy, we found an association with local pain, especially four days after initiating treatment (p = <0.001).

In all laboratory analysis conducted during and at the end of treatment with meglumine antimoniate, there were alterations in renal, hepatic, pancreatic and hematologic tests. There was an association with increased amylase levels (p < 0.005), which reached grade 3 in some cases[39].

### Severe side effects

Three patients experienced serious adverse effects, none of which were related to medications (2 died in combat and 1 was wounded by a knife). These three patients were part of the meglumine antimoniate group.

### Survival analysis

The meglumine antimoniate group experienced significantly fewer failures to treatment (15%) compared to the thermotherapy group (36%) (log rank = 9.6, p = <0.001).

## Discussion

This study followed procedures delineated by the GCP. The sample size was adequate for the study. The follow-up rate was 88% at six months after treatment, which was higher than estimated when calculating the sample size.

In the meglumine antimoniate group, treatment efficacy by protocol and intention-to-treat were 85 and 72%, respectively. These results demonstrate a decrease in the rate of efficacy for this age group, which was reported as 93% during the 1990s [41-43]. This decrease in the efficacy of antimonials could be related, among other factors, to the administration of incomplete doses as a consequence of adherence problems, unavailability of a complete dose to all patients, black market of medications in rural areas and evidence that men also play a role in the transmission of American cutaneous leishmaniasis [29]. There was no statistically significant difference in terms of treatment efficacy with leishmania species.

Thermotherapy had an efficacy of 64 and 58% per protocol and intention-to-treat, respectively; these results are comparable to the findings reported by a study in Kabul, Afghanistan, (CL due to *L. (L) tropica*) in which the efficacy per protocol was 69% [44]. In other study made with patients from other Asian countries (Irán and Kuwait), following the same conditions of application of local heat and where the isolated specie was *L. (L) major*, the intent efficacy of thermotherapy to treat was 48%, two months after treatment ends[45].

With regard to efficiency, we found that meglumine antimoniate is statistically superior to thermotherapy for the treatment of CL in Colombia (p < 0.001), but we also found that meglumine antimoniate has been associated with severe side effects in the musculoskeletal system (myalgia and arthralgia), fever, headaches [4] and toxicity in organs such as the kidney, the pancreas and the hematologic and cardiovascular systems. These effects are not associated with thermotherapy, which only causes local pain four days after the initiation of treatment.

Systemic treatment with pentavalent antimonials for cutaneous leishmaniasis caused by *L. (V) panamensis* and *L. (V) braziliensis* was recommended by the WHO in 1990 under the assumption that such treatment prevents the development of mucosal lesions[46]. However, subsequent studies conducted in Peru demonstrated that mucosal lesions may appear despite the use of these therapeutic schemes. In the case of Colombia, where the predominant parasite species are *L. (V) braziliensis* and *L. (V) panamensis,* the need for all patients to receive systemic treatment of 20 mg Sb^5^/kg/day for 20 days is even more controversial, especially because the incidence of mucosal leishmaniasis is less than 0.5% although it is estimated that, in rural areas far from health centers, only 10% of the population with CL receives complete treatment with antimonials [28-30]. Unpublished data (Dirección de Sanidad del Ejército (DISAN) and Programa de Estudio y Control de Enfermedades Tropicales (PECET)) documented 12 fatalities related to the use of this therapy, which is unacceptable for a form of the disease that is not fatal.

Thermotherapy has been evaluated in several American and Old World countries with efficacy rates of more than 70%. In Colombia, only one report of thermotherapy used to treat CL was made in an epidemic area of *L. (V) guyanensis*. In this region, thermotherapy had an efficacy of 100 and 19% by protocol and intention-to-treat, respectively, although the lack of follow-up in many patients (81%) decreased the efficacy of the study and limited the interpretation of results[47].

The facts mentioned previously led us to ponder the convenience of using local treatments (thermotherapy, paromomicine, cryotherapy, intra-lesion pentavalent antimony) in combination with patient education on how to detect early signs and symptoms of mucosal complications so they can be promptly reported to the physician and addressed with systemic treatment.

The results of the present study show that thermotherapy could be considered a valid alternative treatment due to its efficacy (>60%) and safety as well as because it requires a single session and does not require laboratory monitoring.

Although the equipment required for thermotherapy (ThermoMed) is very expensive and difficult to access in endemic countries due to its cost, the expenses related to conventional treatment overweigh the costs of Thermomed.

With the goal of increasing its efficacy, we propose a subsequent clinical trial to evaluate new schemes of application of local treatment with thermotherapy; for example, patients may undergo 2–3 sessions or a combination of local treatments with a low systemic dose.

## Conclusions

Although the efficacy rate of meglumine antimoniate was greater than that of thermotherapy for the treatment of cutaneous leishmaniasis, the side effects were also greater. Those factors, added to the increased costs, the treatment adherence problems and the progressive lack of therapeutic response, make us consider thermotherapy as a first line treatment for cutaneous leishmaniasis.

The thermotherapy is also a valid alternative in patients with renal, hepatic and cardiac illness who cannot receive systemic therapy. It is important to mention that thermotherapy should not be applied near mucosal areas.

## Ethical approval

The protocol was approved by the Bioethics Committee for Human Research of “Sede de Investigacion Universitaria” (CBEIH-SIU) from the University of Antioquia and by the Ethics Committee of the General Health Management of the Colombian Army and and carried out according to international norms of good clinical practice

## Consent informed

Before deciding to participate in the research, All patients signed a informed consent form in the presence of two witnesses.

## Abbreviations

CL, Cutaneous leishmaniasis; ML, Mucosal leishmaniasis; VL, Visceral leishmaniasis; CTCAE, Common Terminology Criteria for Adverse Events; CI, Confidence Interval; PECET, Programa de Estudio y Control de Enfermedades Tropicales; DISAN, Dirección de Sanidad del Ejército.

## Competing interest

The authors declare that they have no competing interests.

## Authors' contributions

LL coordinated and conducted the field work, participated in the design and reporting of the study; MR and MV carried out field work (inclusion and follow-up of volunteers); IV participated in the design, execution and reporting of the study. All authors read and approved the final manuscript. IV is guarantor of the paper.

## Authors' information

LL MSc. Clinical Trials Coordinator PECET. MR Dermatologist, Colombia Army. MV Dermatologist, Colombia Army. IV MD MSc PhD. Director PECET, Professor University of Antioquia - Colombia, member of the "Data Management Team" TDR/WHO, Clinical Monitor TDR/WHO.
