# Erratum to: Thermotherapy. An alternative for the treatment of American cutaneous leishmaniasis

**DOI:** 10.1186/s13063-017-2092-3

**Published:** 2017-09-01

**Authors:** Liliana López, Martha Robayo, Margarita Vargas, Iván D. Vélez

**Affiliations:** 1 Program for the Study and Control of Tropical Disease, University of Antioquia, Carrera 53 #61-30, Medellín, Colombia; 2 Dirección de Sanidad, DISAN, Colombia Army, Bogotá, Colombia

## Erratum

The original publication [1] misses 4 tables and 1 figure which should have been available as additional files. The missing additional files have been included with this erratum as well as a list with description which can be found below.

Table S1: As shown in Additional file 1, both groups had similar demographic, clinical and parasitologic characteristics.

Table S2: By intention-totreat, the definitive healing rate in the thermotherapy group was 58.5% (95% IC 49–66) and 72% (95% IC 78– 92) in the Meglumine antimoniate group, as shown on Additional file 2.

Table S3 and the *Leishmania* species identified. There was also no association between treatment and other variables such as number, location and lesion type or with the geographical area of Colombia where the infection occurred (Additional file 3).

Table S4: Additional file 4 shows the systemic and local side effects found in this study.

Figure S1: Additional file 5 Flow diagram.

## Additional files

Additional file 1: Table S1. Baseline characteristics of the volunteers. (PDF 198 kb)

Additional file 2: Table S2. Efficacy of meglumine antimoniate and thermotherapy. Analysis by protocol and intention-to-treat. (PDF 174 kb)

Additional file 3: Table S3. Efficacy of the Meglumine Antimoniate and Thermotherapy stratified by parasite species, anatomic location, number and type of lesions and geographic region of the infection. (PDF 188 kb)

Additional file 4: Table S4. Incidence and Relative Risk of local and systemic side effects at mid-treatment and at the end of treatment. (PDF 222 kb)

Additional file 5: Figure S1. Diagram of the volunteers who were part of the study. (PDF 89 kb)
