# Supplementary material for: Erratum to: Thermotherapy. An alternative for the treatment of American cutaneous leishmaniasis
Source: Trials. 2017 Sep 1;18:408. doi: 10.1186/s13063-017-2092-3 (PMC5579890; doi:10.1186/s13063-017-2092-3)
Supplement: Supplementary file 1 — Baseline characteristics of the volunteers. (PDF 198 kb) [file 13063_2017_2092_MOESM1_ESM.pdf]

Table 1. Baseline characteristics of the volunteers

Characteristic		Meglumine Antimony n=143	Thermotherapy n= 149	P value[*]
Median age in years (range)		23 (19 - 38)	23 (19 - 39)	0,[†]
Race (%)	White	17 (12)	22 (14)	0,25
	Black	6 (4)	13 (9)	
	Mestizo	115 (80)	104 (70)	
	Mulatto	5 (4)	10 (7)	
Median weight in kg (range)		65 (50 - 96)	65 (42 -104)	0,[†]
History of Leishmaniasis (%)	Yes	52 (37)	67 (45)	0,79
	No	91 (64)	82 (55)	
Geographic area of infection	Northeast	17 (12)	16 (11)	0,91
	Southeast	126 (88)	133 (89)	
Number of lesions (%)[‡]	1	97(68)	115 (77)	0,14
	2 or more	46(32)	34 (23)	
Lesion type (%)	Nodule	14 (6)	19 (11)	0,04
	Ulcer	223 (94)	161 (89)	
Anatomic localization of lesions	Upper body	118 (83)	123 (83)	0,68
	Lower body[§]	25 (17)	26 (17)	
Median evolution time in Days (range)		60 (6 – 210)	85 (10 - 690)	0,6[†]
Species (%)	*L. panamensis*	32 (38)	24 (29)	0,27
	L. braziliensis	52 (62)	59 (71)	

[*] Chi-square test
[†] Kruskall Wallis test

[‡] According to number of lesions
[§] In this category, we included 18 volunteers who presented lesions in the upper and lower part of the body; 8 were from the Meglumine Antimoniate group, and 10 were from the Thermotherapy group.
