# Supplementary material for: Erratum to: Thermotherapy. An alternative for the treatment of American cutaneous leishmaniasis
Source: Trials. 2017 Sep 1;18:408. doi: 10.1186/s13063-017-2092-3 (PMC5579890; doi:10.1186/s13063-017-2092-3)
Supplement: Supplementary file 2 — Efficacy of meglumine antimoniate and thermotherapy. Analysis by protocol and intention-to-treat. (PDF 174 kb) [file 13063_2017_2092_MOESM2_ESM.pdf]

Table 2. Efficacy of meglumine antimoniate and thermotherapy. Analysis by protocol and intention-to-treat.

Analysis	Meglumine Antimoniate		Thermotherapy		P Value*
	Cure / Total	Efficacy (%) IC 95%	Cure / Total	Efficacy (%) IC 95%	
Intention-to-treat	103/143	72 (64 – 80)	86/149	58 (49 – 66)	0,01
By protocol	103/121	85 (78 – 92)	86/134	64 (55 – 73)	<0,001

* Square Chi test
