# Supplementary material for: Erratum to: Thermotherapy. An alternative for the treatment of American cutaneous leishmaniasis
Source: Trials. 2017 Sep 1;18:408. doi: 10.1186/s13063-017-2092-3 (PMC5579890; doi:10.1186/s13063-017-2092-3)
Supplement: Supplementary file 3 — Efficacy of the Meglumine Antimoniate and Thermotherapy stratified by parasite species, anatomic location, number and type of lesions and geographic region of the infection. (PDF 188 kb) [file 13063_2017_2092_MOESM3_ESM.pdf]

Table 3. Efficacy of the Meglumine Antimoniate and Thermotherapy stratified by parasite species, anatomic location, number and type of lesions and geographic region of the infection

Characteristic	Efficacy/ volunteers (%)	P*	Efficacy/ volunteers (%)	P*
	Meglumine Antimoniate		Thermotherapy	
Overall efficacy	103/143 (72)	-	86/149 (58)	-
**Species**<br>*L. panamensis*<br>*L. braziliensis*	 23/32(72)<br>34/52(65)	 0,5	 14/24 (58)<br>31/59(53)	 0,6
**Number of lesions**<br>1<br>2 or more	 72/97 (74)<br>31/46 (67)	 0,4	 70/115 (61)<br>17/34 (50)	 0,3
**Anatomic location**<br>Upper body<br>Lower body†	 87/118 (73)<br>16/25 (64)	0,3	73/123 (59)<br>14/26 (54)	0,6
**Geographic region of the infection**<br>Northeast<br>Southeast	9/17 (53)<br>94/126 (75)	0,06	6/16 (38)<br>81/133 (60)	0,08

* Fisher exact test

† In this category, we included 18 volunteers who presented lesions in the upper and lower part of the body; 8 were from the Meglumine Antimoniate group, and 10 were from the Thermotherapy group
