# Supplementary material for: Erratum to: Thermotherapy. An alternative for the treatment of American cutaneous leishmaniasis
Source: Trials. 2017 Sep 1;18:408. doi: 10.1186/s13063-017-2092-3 (PMC5579890; doi:10.1186/s13063-017-2092-3)
Supplement: Supplementary file 4 — Incidence and Relative Risk of local and systemic side effects at mid-treatment and at the end of treatment. (PDF 222 kb) [file 13063_2017_2092_MOESM4_ESM.pdf]

Table 4. Incidence and Relative Risk of local and systemic side effects at mid-treatment and at the end of treatment

	Test/Event	Mid-treatment Test or Event/ Total volunteers (%)		RR (IC 95%)	End of treatment Test or Event/ Total volunteers (%)		RR (IC 95%)
		MA	Thermotherapy		MA	Thermotherapy	
Side Effects	Fever	11/119(9,24)	4/124(3,22)	2,87 (0,94 – 8,75)	29/131(22,13)	4/132(3)	7,31 (2,64 – 20,2)
	Myalgia	12/119(10,1)	3/124(2,41)	4,17 (1,21 – 14,40)	67/131(51,14)	4/132(3)	16,9 (6,34 – 44,9)
	Arthralgia	17/119(14,28)	3/124(2,41)	5,9 (1,78 – 19,63)	65/131(49,61)	3/132(2)	21,8 (7,04 – 67,7)
	Headache	17/119(14,28)	10/124(8,1)	1,77 (0,85 – 3,71)	52/131(39,69)	13/132(10)	4,03 (2,31 – 7,04)
	Vomit	3/119(2,52)	3/124(2,41)	1,04 (0,21 – 5,06)	16/131(12,21)	2/132(2)	8,06 (1,89 – 34,4)
	Nauseas	4/119(3,36)	5/124(4,03)	1,39 (0,32 – 6,08)	27/131(20,61)	4/132(3)	6,8 (2,45 – 18,9)
	Anorexia	8/119(6,72)	5/124(4,03)	1,67 (0,56 – 4,95)	45/131(34,35)	6/132(5)	7,56 (3,34 – 17,1)
	Diarrhea	0/119 (0)	0/124 (0)	-	2/131(1,53)	1/132(1)	2,02 (0,18 – 21,3)
	Abdominal pain	0/119 (0)	0/124(0)	-	2/131(1,53)	0/132 (0)	-
	Lesion pain	3/119(2,52)	27/124(22)	0,12 (0,04 – 0,37)	16/131(12,21)	18/132(14)	0,9 (0,48 – 1,68)
	Lesion infection	2/119(1,68)	9/124(7)	0,23 (0,05 – 1,05)	5/131(3,82)	11/132(8)	0,43 (0,15 – 1,19)
	Blisters on lesion	0/119 (0)	4/124(3)	-	2/131(1,53)	5/132(4)	0,4 (0,08 – 2,01)
	Pruritus	0/119 (0)	0/124 (0)	-	1/127 (0,78)	4/125 (3)	0,25 (0,03 – 2,17)
Blood Chemistry	↑ BUN	4/108(3,7)	1/83(1)	3,07 (0,35 – 26,99)	1/116(0,86)	2/80(3)	0,34 (0,03 – 3,74)
	Grade 1[†]	3/108(2,77)	1/83(1)	2,31 (0,24 – 21,77)	1/116(0,86)	2/80(3)	0,34 (0,03 – 3,74)
	Grade 2	1/108(0,93)	0/83	-	0/116 (0)	0/80 (0)	-
	↑ AST	1/107(0,93)	1/93(1)	0,87 (0,06 – 13,7)	1/116(0,86)	2/75(2,67)	0,34 (0,03 – 3,74)
	Grade 1[‡]	1/107(0,93)	1/93(1)	0,87 (0,06 – 13,7)	1/116(0,86)	2/75(2,67)	0,34 (0,03 – 3,74)
	↑ ALT	3/110(2,72)	1/93(1)	2,54 (0,27 – 23,98)	20/112(17,9)	5/73(6,84)	2,61 (1,02 – 6,64)
	Grade 1[‡]	2/110(1,81)	1/93(1)	1,69 (0,16 – 18,35)	19/112(17)	5/73(6,84)	2,48 (0,97 – 6,34)
	Grade 2[‡´]	1/110(0,9)	0/93(0)	-	1/112(0,89)	0/73 (0)	-
	↑ Amylase	29/105(27,6)	6/87(7)	3,91 (1,7 – 8,98)	24/109(22)	7/70(10)	2,2 (1,0 – 4,84)
	Grade 1[§]	18/105(17,1)	5/87(6)	2,98 (1,15 – 7,71)	19/109(17,4)	6/70(8,57)	2,03 (0,85 – 4,84)
	Grade 2[§´]	6/105(5,71)	0/87(0)	-	1/109(0,9)	0/70(0)	-
	Grade 3[§´´]	5/105(4,76)	1/87(1)	4,14 (0,49 – 34,8)	4/109(3,7)	1/70(1,42)	2,57 (0,29 – 22,51)
	↓Hemoglobin	-	-	-	5/111(4,5)	1/78(1,28)	3,51 (0,42 – 29,49)

[‡] >ULN – 2,5xULN

[§] ULN – 1,5xULN

[§´] >1,5 – 2,0xULN

[§´´] >2,0 – 5,0xULN

**Hematology**	Grade1[‖]	-	-	-	5/111(4,5)	1/78(1,28)	3,51 (0,42 – 29,49)
	↓ RBC	14/100(14)	4/82(5)	2,87 (0,98 – 8,39)	7/107(6,54)	1/75(1,33)	4,91 (0,62 – 39,06)
	Grade 1[¶]	14/100(14)	4/82(5)	2,87 (0,98 – 8,39)	7/107(6,54)	1/75(1,33)	4,91 (0,62 – 39,06)
	↓ Leukocytes	5/110(4,54%)	0/96 (0)	-	2/115(1,73)	0/79(0)	-
	Grade 1[**]	5/110(4,54%)	0/96 (0)	-	2/115(1,73)	0/79(0)	-
	↓ Platelets	1/110(0,91%)	0/96 (0)	-	1/114(0,87)	1/79(1,27)	0,69 (0,04 – 10,92)
	Grade 1[††]	1/110(0,91%)	0/96 (0)	-	1/114(0,87)	1/79(1,27)	0,69 (0,04 – 10,92)

‖ <LLN – 10,0 g/dL
¶ <LLN – 3,0x10^{12}/L
** <LLN – 3,0x10^{9}/L
†† <LLN – 75,0x10^{9}/L
