# Supplementary figures and images for: Erratum to: Thermotherapy. An alternative for the treatment of American cutaneous leishmaniasis
Source: Trials. 2017 Sep 1;18:408. doi: 10.1186/s13063-017-2092-3 (PMC5579890; doi:10.1186/s13063-017-2092-3)

**Figure 1. Diagram of the volunteers who were part of the study**

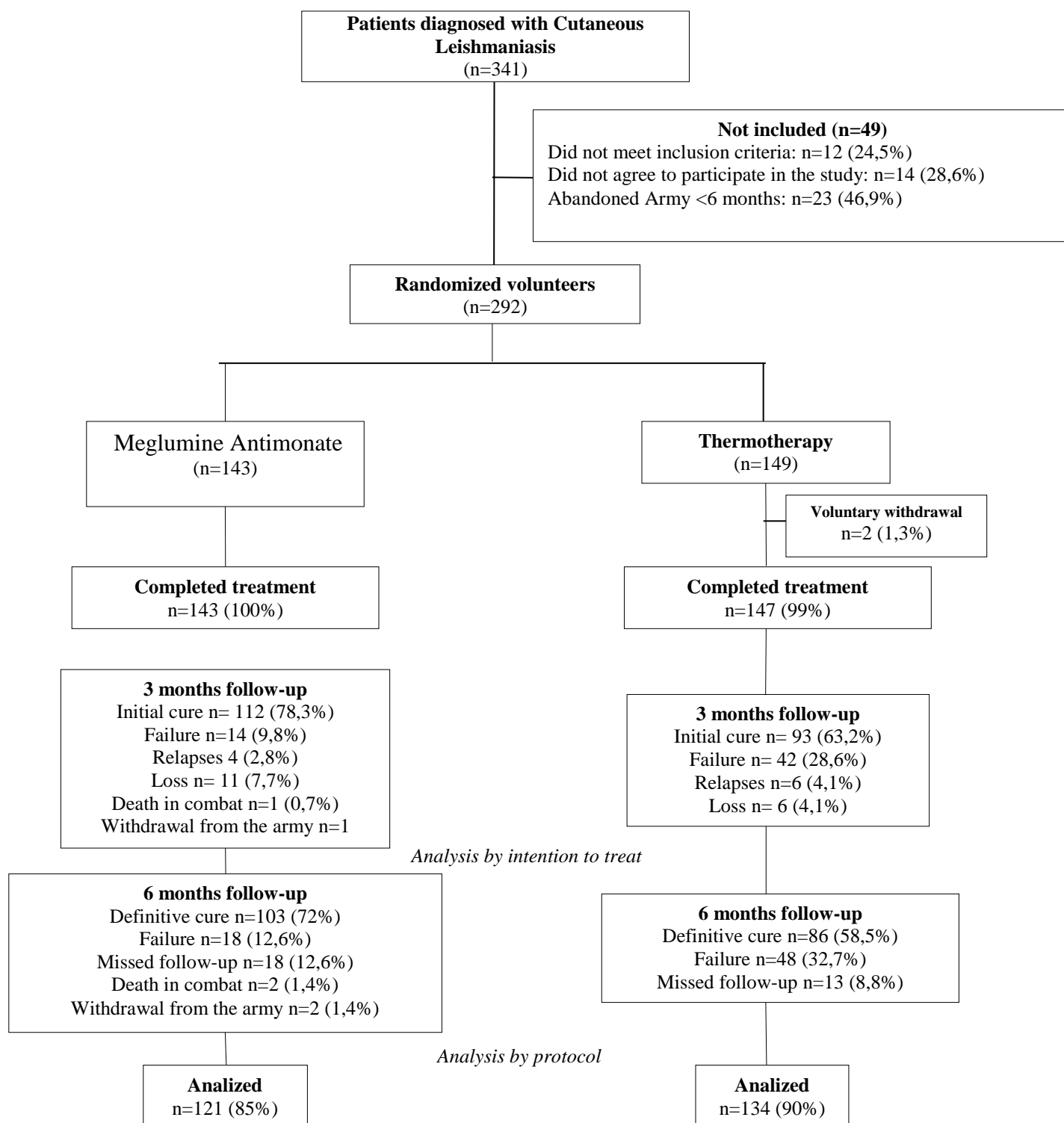

Supplement: Supplementary file 5 — Diagram of the volunteers who were part of the study. (PDF 89 kb) [file 13063_2017_2092_MOESM5_ESM.pdf]
